# Muddled mechanisms: recent progress towards antimalarial target identification

**DOI:** 10.12688/f1000research.9477.1

**Published:** 2016-10-13

**Authors:** Rachel L. Edwards, Audrey R. Odom John

**Affiliations:** 1 Department of Pediatrics, Washington University School of Medicine, St. Louis, MO, USA; 2 Department of Molecular Microbiology, Washington University School of Medicine, St. Louis, MO, USA

**Keywords:** Malaria, Plasmodium falciparum, Asia, therapy

## Abstract

In the past decade, malaria rates have plummeted as a result of aggressive infection control measures and the adoption of artemisinin-based combination therapies (ACTs). However, a potential crisis looms ahead. Treatment failures to standard antimalarial regimens have been reported in Southeast Asia, and devastating consequences are expected if resistance spreads to the African continent. To prevent a potential public health emergency, the antimalarial arsenal must contain therapeutics with novel mechanisms of action (MOA). An impressive number of high-throughput screening (HTS) campaigns have since been launched, identifying thousands of compounds with activity against one of the causative agents of malaria, *Plasmodium falciparum*. Now begins the difficult task of target identification, for which studies are often tedious, labor intensive, and difficult to interpret. In this review, we highlight approaches that have been instrumental in tackling the challenges of target assignment and elucidation of the MOA for hit compounds. Studies that apply these innovative techniques to antimalarial target identification are described, as well as the impact of the data in the field.

## Introduction

Due to the concerted efforts to reduce malaria morbidity and mortality, it is estimated that since 2000, more than 663 million clinical cases and 6.2 million deaths have been averted in sub-Saharan Africa ^1^. The drastic reduction in malaria burden is largely due to the implementation of infection control measures, including the adoption of the highly effective artemisinin-based combination therapies (ACTs). In Africa alone, ACTs save approximately 100,000 lives each year ^2^. Despite these successes, malaria remains a major threat to public health. Annually, 3.2 billion people are at risk of infection and more than 400,000 die, with young children and pregnant women being disproportionately affected ^3^. While ACTs remain the cornerstone for global malaria treatment, recent reports indicate that current regimens are failing ^4– 8^. Thus, new classes of antimalarial compounds are desperately needed to combat emerging and existing drug-resistant parasites, if the progress made in the last decade is not to be undone ^9^.

To this end, over 6 million compounds have been screened against *Plasmodium falciparum*, the etiological agent responsible for the bulk of malaria deaths ^10^. Initially, high-throughput screening (HTS) campaigns concentrated on the intraerythrocytic stage of *P. falciparum*, which resulted in an unprecedented number of hits with the potential to treat the symptomatic stage of the disease ^11– 17^. Antimalarials that target the liver stages and the asymptomatic gametocyte stages will be critical as priorities shift from treatment towards local elimination. Thus, recent endeavors have focused on screening agents against these more tenacious stages of the *P. falciparum* life cycle ^18– 26^. As a result of the collaborative efforts between academia and the pharmaceutical industry, more than 25,000 compounds with half-maximal inhibitory concentration (IC _50_) activity ≤1 μM against *P. falciparum* now await target identification and further characterization ^10^.

Estimates suggest that 7% of drugs approved by the US Food and Drug Administration (FDA) lack a defined target, and approximately 18% lack a definitive mechanism of action (MOA) ^27– 29^. While assigning the target and MOA of a compound are clearly not essential for its development, this information is often crucial in hit-to-lead optimization. For example, target identification informs medicinal chemistry to improve selectivity and/or pharmacokinetic and toxicity profiles, without sacrificing potency ^30^. A molecular understanding of compound action may also direct dosing, aid in partner drug selection, and assist with drug resistance surveillance ^31, 32^. Finally, once a target has been identified and validated, inhibitors may be instrumental in probing essential parasite biology ^32^.

Elucidating the molecular targets responsible for the phenotypic effects observed in cell-based assays is often one of the most challenging and time-consuming steps in drug discovery. For *P. falciparum*, MOA assignment for first-in-class drugs has traditionally been quite difficult, as almost 50% of the genome lacks annotation, transcriptional profiling has had varying results, and heterologous protein expression remains problematic ^32– 35^. Moreover, the majority of known antimalarial agents have pleiotropic effects and exhibit polypharmacology, a likely outcome for many of the identified hits that will further complicate target assignment ^36– 38^. Often, tedious biochemical, genetic, and cellular studies are needed to deeply understand the MOA of a compound, as demonstrated by many attempts to identify the targets and biological effects of the elusive antimalarials atovaquone and artemisinin ^39– 41^. To overcome these barriers, a number of target deconvolution strategies have been developed, including resistance screening, transcriptional profiling, proteomic ** analysis, and metabolic analysis ^32, 42^. In this brief review, we describe the recent advances in experimental target identification in *P. falciparum* and present examples that exemplify each method. Of note, *in silico* approaches of target assignment have been covered separately in recent reviews ^43– 45^.

## Genetic approaches of target identification

### Resistance screening

To discern the target and the MOA of a novel antimalarial agent, one method that has been commonly employed is *in vitro* evolution of resistant parasites. Drug pressure is applied to cloned *P. falciparum* cultures either at a single concentration or in a stepwise fashion. In a recent large study to develop resistant mutants against many novel antimalarials, *de novo* resistance appears to occur rapidly in more than half of such attempts ^9, 32, 46^. Resistant parasites are then cloned, and the genomic DNA is isolated and analyzed by next-generation sequencing to identify genetic changes associated with resistance ^46^. The genomes of the parental and mutant parasite lines are compared to identify single nucleotide polymorphisms (SNPs) and copy number variants (CNVs) ^46^. Propagation of drug-resistant *P. falciparum* has successfully assigned a number of known and proposed antimalarial targets, including 1-deoxy-D-xylulose 5-phosphate reductoisomerase ^47^, cytochrome *bc*_1_^48– 50^, apicoplast-localized and cytoplasmic isoleucyl tRNA synthetase ^51^, signal peptide peptidase ^52^, lysyl-tRNA synthetase ^53^, dihydroorotate dehydrogenase ^54^, prolyl-tRNA synthetase ^55^, and the P-type ATPase 4 (PfATP4) ^9, 56– 61^. Remarkably, genetic changes in PfATP4 have been found to associate with resistance to multiple antimalarial chemotypes (spiroindolones, pyrazoles, dihydroisoquinolones, MMV722, MMV011567, and MMV007275) ^9, 56– 61^. Currently, the reason for this commonly observed, PfATP4-associated resistance is unclear, but several mechanisms have been proposed ^61^.

Caution is required when assigning compound MOA based on *in vitr*o resistance selection and associated genetic changes. Multiple examples in malaria parasites and other organisms have shown that mutations in genes distinct from the molecular target may yet confer resistance. For example, mutations in the *P. falciparum* multi-drug resistance transporters, such as MDR1, mediate resistance to multiple classes of antimalarials due to compound transport. Resistance alleles may reveal related parasite biology, as in work by Guggisberg *et al*., which demonstrated that a mutation in a metabolic regulator, HAD1, confers resistance to fosmidomycin (FSM) due to changes in intracellular metabolite levels ^62^. These genetic changes would arguably have been far more difficult to interpret if the target of FSM (PfDXR) had not already been well established. While the generation of resistant mutants has been instrumental for target validation in *P. falciparum*, neither genetic nor chemical methods alone can definitively conclude that an enzyme, metabolic pathway, or cellular function is indeed the target; thus, complementary approaches must be utilized ^63^.

### Chemogenomic profiling

To date, global chemical-genetic methods for drug target identification have been relatively underutilized in *P. falciparum.* Chemogenomic profiling represents a powerful tool that deduces MOA by comparing alterations in drug fitness profiles within a panel of mutants ^64, 65^. In 2015, the first chemogenomic screen of *P. falciparum* was performed with a library of 71 random *piggyBac* transposon insertion mutants and 53 antimalarial drugs and metabolic inhibitors ^64^. The antimalarial drug sensitivities were monitored in the mutant parasite lines, and thus the chemogenomic interactions and the relationships between drug pairs were discerned ^64^. Interestingly, a cluster of seven mutants were identified that were sensitive to artemisinin, including one with a mutation in the K13-propeller gene that is associated with resistance ^64, 66, 67^. In a second study by Aroonsri *et al*., reverse-genetic chemogenomic profiling was used to uncover novel antimalarial agents that target dihydrofolate reductase-thymidylate synthase (DHFR-TS) ^65^. By screening a small compound library, two novel DHFR-TS inhibitors (MMV667486 and MMV667487) were identified with activity against blood-stage *P. falciparum*. Presumably, additional DHFR-TS inhibitors could be discovered by screening larger, more diverse chemical libraries ^65^. Together, the aforementioned studies demonstrate the utility of chemical-genetic approaches in target assignment and pave the way for additional chemogenomic profiling in *P. falciparum*.

## Target predictions via transcriptional analysis

Monitoring the global changes in gene expression may reveal regulatory and metabolic networks affected by drug treatment ^68^. Thus, expression data may help elucidate the MOA of a drug and facilitate the characterization of unannotated genes ^68^. Unfortunately, transcriptional profiling of drug-treated *P. falciparum* has been met with mixed results ^69^. Several studies reported that expression changes are limited following antimalarial treatment, suggesting that malaria parasites are transcriptionally hard-wired ^33– 35^. Conversely, other studies have shown that chemical perturbations produce transcriptional responses in the expected target biological pathways ^70– 73^.

Recent work from Siwo *et al.* profiled the effect of 31 chemically and functionally diverse small molecules on *P. falciparum* ^74^. By building a series of controls into their study design and by incorporating several normalization steps into their analysis, the transcriptional responses induced by each drug were successfully disentangled ^74^. This novel approach not only identified transcriptional changes in expected target pathways but also provided evidence that artemisinin targets cell cycle and lipid metabolism, consistent with previous data ^74–84^. Further, the MOA were predicted for several HTS-selected compounds by correlating the connections identified in the small molecule-Gene Ontology (GO) network with the functions of genes located in their quantitative trait locus (QTL) ^13, 74^. Importantly, the study explains why previous gene expression studies failed to tease out drug-specific responses and demonstrates that transcriptional profiling can capture the complexity of drug effects and accurately assign drug targets.

## Proteomic approaches of target assignment

Despite the trove of information that can be gleaned from using DNA and RNA analyses to identify drug targets, genomic methods alone are insufficient to capture the total cellular effects of a given antimalarial ^85^. *P. falciparum* has approximately 5,300 protein-encoding genes ^86, 87^. In theory, monitoring the global proteomic changes following drug treatment may inform on the function, expression, localization, interacting partners, and regulation of every protein, thus providing clues to compound MOA ^85^. Conventional proteomic methods have been used for drug target identification in *P. falciparum*. For example, mass spectrometry (MS) was used to analyze alterations in the parasite proteome following chloroquine or artemisinin treatment ^88^, two-dimensional gel electrophoresis (2-DE) and tandem MS were used to identify protein changes in chloroquine-treated *P. falciparum* ^89^, and finally isobaric tags for absolute and relative quantification (iTRAQ) and two-dimensional fluorescence gel electrophoresis (2D-DIGE) were used to monitor protein expression in doxycycline-treated parasites ^90^. More recently, 2-DE and tandem MS identified proteins differentially expressed following treatment with quinine, mefloquine, or the natural product diosgenone ^91^. While these traditional methods can provide useful information regarding global proteomic changes, it should be mentioned that a major limitation of these techniques is that low-abundance proteins may be outside the detection limits.

### Chemical proteomics

The emerging field of chemical proteomics uses synthetic chemistry to design and generate probes to identify small-molecule–protein interactions ^92, 93^. This global proteomic approach detects interacting partners via MS-based affinity chromatography, and interactions are then mapped to signaling and metabolic pathways ^92^. Applications include characterizing drug targets, deducing protein function, and uncovering off-target effects ^92, 93^. Chemical proteomic techniques are separated into two classifications: (1) activity-based protein profiling (ABPP), which monitors enzyme activities, or (2) compound-centric approaches, which reveal direct molecular interactions between compounds and targets ^92, 94^. Both methods provide broad, unbiased analyses and have been successfully applied to antimalarial discovery research.

A typical chemical strategy is synthesis of compound analogs to incorporate a “click” handle to facilitate drug target identification and validation in *P. falciparum* ^95^. For example, a bifunctional compound based on the clinical candidate albitiazolium was synthesized that was photoactivatable and taggable ^96^. MS identified a discrete list of potential drug targets in *P. falciparum*, while bioinformatic and interactome analyses were used to predict protein functions ^96^. As albitiazolium inhibits phospholipid metabolism, most of the target proteins are involved in lipid metabolic activities ^96^. A number of surprising targets were also uncovered, such as proteins involved in vesicular budding and transport functions, thereby demonstrating the utility of the method ^96^. Further, in work by Wang *et al*., an artemisinin analog was engineered with a “clickable” alkyne tag that was coupled with either a biotin moiety for protein target identification or a fluorescent dye that would enable the activation mechanism of the drug to be monitored ^36^. This dual chemical proteomics approach identified 124 putative direct targets of artemisinin, 33 of which had been proposed previously as antimalarial drug targets ^36^. In a subsequent study, a panel of activity-based probes was generated that incorporated the endoperoxide scaffold of artemisinin as a warhead to alkylate the molecular targets in *P. falciparum* ^97^. Tagged proteins were then isolated and identified by liquid chromatography (LC)-MS/MS ^97^. Importantly, alkylated targets were identified in glycolytic, hemoglobin degradation, antioxidant defense, and protein synthesis pathways, supporting the promiscuous activity of artemisinin ^36, 74, 97^.

Methods of target identification have been developed that do not rely on chemical modification of the investigative compound, including the cellular thermal shift assay (CETSA), drug affinity responsive target stability (DARTS), stability of proteins from rates of oxidation (SPROX), and thermal proteome profiling (TPP) ^98^. While successful identification is largely dependent on the abundance of the drug target, these methods are less time consuming, avoid diminishing or altering drug activity, and can capture both the on- and off-target proteomic effects on a global scale ^98^. Recently, a DARTS assay was conducted to identify targets for Torin 2, a lead compound with low nM activity against *P. falciparum* gametocytes ^99^. Three gametocyte proteins (phosphoribosylpyrophosphate synthetase, PF3D7_1325100; aspartate carbamoyltransferase, PF3D7_1344800; and a transporter, PF3D7_0914700) were identified as putative targets for Torin 2, demonstrating the utility of label-free, chemical proteomic approaches in *P. falciparum* ^99^. We anticipate that due to their unbiased nature and versatility, future antimalarial drug discovery ventures will incorporate comparable technologies into the pipeline, thereby accelerating target assignment.

## Target identification through metabolite analysis

Small metabolic perturbations can have a dramatic impact on critical cellular processes such as cell division, differentiation, and stress response pathways. Accordingly, a number of antimalarial agents in clinical development target metabolic enzymes, including the enzymes of the electron transport chain (ELQ-300, GSK932121, DSM265) ^100– 102^, the methylerythritol phosphate (MEP) pathway (FSM, MMV008138) ^103– 106^, the folate biosynthetic pathway (P218) ^107^, and phosphoinositide metabolism (MMV390048) ^108^. It is predicted that many of the active compounds uncovered by HTS projects will also have metabolic targets. To expedite target assignment of the novel drugs and drug scaffolds identified via HTS projects, metabolomic strategies are increasingly being incorporated into the drug-screening pipeline.

### Targeted metabolite profiling

When candidate targets are known, researchers may focus their efforts by analyzing only a subset of metabolites; however, this requires prior knowledge of the enzymes, their kinetics and end products, and the established pathways in which they participate ^109^. Targeted methodologies facilitate the enrichment of low-abundance analytes and incorporate the use of internal standards to permit quantitative metabolite analysis ^109^. Such metabolite profiling has been successfully employed to identify and validate a number of antimalarial drug targets. Notable examples include identification of targets for the novel quinolone CK-2-68 (NADH:ubiquinone oxidoreductase and cytochrome *bc*_1_), eflornithine (ornithine decarboxylase), MDL73811 (AdoMetDC), and a second target for the MEP pathway inhibitor FSM ^70, 104, 110, 111^.

More recently, targeted metabolite analysis was also used to characterize the enzymes of the NAD ^+^ salvage pathway in *P. falciparum* ^112^. By tracing ^13^C-labeled compounds via mass spectrometry, O’Hara *et al*. demonstrated that parasites scavenge exogenous niacin from their host ^112^. Nicotinate mononucleotide adenylyltransferase (PfNMNAT) enzyme within the pathway was required for NAD ^+^ metabolism and, further, the *P. falciparum* enzyme was similar to bacterial NMNATs ^112^. Parasites were then screened against a panel of bacterial NMNAT inhibitors and a compound with a minimum inhibitory concentration (MIC) <1 μM against ring-stage *P. falciparum* was identified, validating NMNATs as an inhibitable drug target ^112^.

### Metabolomics

In contrast, untargeted metabolite analysis may be used to perform a global survey of the metabolic fluctuations induced by drug treatment. Metabolomic-based technologies measure the small molecule repertoire of the cell in response to a stimulus, such as drug treatment ^111, 113^. The resulting metabolic signature reveals the metabolites and pathways that are perturbed and, accordingly, assists with target identification ^30^. Moreover, metabolomic strategies are especially useful in characterizing drugs that impact multiple targets and identifying any off-target drug effects ^114^. It is important to note that extraction efficiencies, separation methods, and sample degradation can greatly influence the chemical diversity and concentrations of the metabolites present ^114, 115^. In addition, many metabolite features within a sample will remain unassigned, as current databases contain a small number of identified compounds ^114, 115^. While experimental techniques and metabolite identification methods have greatly advanced in recent years, untargeted metabolomic approaches for determining MOA remain limited.

Metabolomics in *P. falciparum* is arguably in its infancy. However, two new studies have demonstrated the utility of unbiased metabolite analysis in drug discovery. First, a dual gas chromatography (GC)-MS and LC-MS approach was used to map the metabolic changes induced by known antimalarial agents in blood-stage parasites ^31^. Although the MOA was confirmed for a number of clinical agents, metabolomics data uncovered that dihydroartemisinin (DHA) not only disrupts hemoglobin catabolism but also perturbs pyrimidine biosynthesis ^31^. The authors then used their untargeted MS approach to characterize a novel antimalarial, Torin 2, as an inhibitor of hemoglobin catabolism ^31^.

In a second study, the total lipid landscape was surveyed during *P. falciparum* blood-stage development ^116^. In addition to identifying ten new lipid classes and confirming the essentiality of the prominent lipid classes in the parasite, the authors tested a panel of compounds known to target lipid metabolisms ^116^. Several inhibitors had low micromolar activity against asexual *P. falciparum* (CAY10499, Orlistat, DL- *threo*-1-phenyl-2-palmitoylamino-3-morpholino-1-propanol, GW4869, epoxyquinone, and N,N-dimethylsphingosine), suggesting that the lipid metabolic enzymes are possible drug targets ^116^. Taken together, these two influential studies demonstrate that integration of metabolomics into the drug discovery pipeline will be crucial for accelerating target assignment.

## Concluding remarks

In the past decade, the research and development portfolio of antimalarial agents has expanded, with approximately 20 new drugs now at various stages of development ^74^. Considerable time, effort, and cooperation between academia and industry have led to the identification of 25,000 hit compounds, many of which may prove to be successful therapeutics ^10^. Now, thousands of compounds lie in wait, as the more difficult and time-consuming task of hit-to-lead optimization must be launched, independently, for each potential candidate. Although not essential, target identification not only helps prioritize hits but also guides medicinal chemists in their quest to improve potency and pharmacokinetic properties ^32^. Target assignment for a novel drug demands that innovative approaches are used to reveal clues of its MOA. Drug candidate attrition is inevitable and resistance development is expected ^10^. As such, the drug discovery pipeline should be flooded with candidates that represent a broad spectrum of MOAs.

A collection of 400 diverse compounds with antimalarial activity was assembled by the Medicines for Malaria Venture into the Malaria Box, a resource that was made available free of charge in the hopes of catalyzing drug discovery research ^14^. More than 250 Malaria Boxes were distributed between 2011 and 2015, and large amounts of data have been deposited into the public domain ^117^. Recently, a meta-analysis was performed on the 291 Malaria Box screens conducted by 55 different research groups ^118^. Aggregated data from all biochemical and cellular assays revealed likely MOA for only 135 (34%) of the compounds ^118^. Therefore, we predict that a multi-pronged attack is almost certainly required for target assignment and MOA identification in the *P. falciparum* drug discovery pipeline.
